# Data on evolutionary hybrid neural network approach to predict shield tunneling-induced ground settlements

**DOI:** 10.1016/j.dib.2020.106432

**Published:** 2020-10-21

**Authors:** Kun Zhang, Hai-Min Lyu, Shui-Long Shen, Annan Zhou, Zhen-Yu Yin

**Affiliations:** a Department of Civil Engineering, School of Naval Architecture, Ocean, and Civil Engineering, Shanghai Jiao Tong University, Shanghai 200240, China; b MOE Key Laboratory of Intelligent Manufacturing Technology, College of Engineering, Shantou University, Shantou, Guangdong 515063, China; c Civil and Infrastructure Discipline, School of Engineering, Royal Melbourne Institute of Technology (RMIT), Victoria 3001, Australia; d Department of Civil and Environmental Engineering, the Hong Kong Polytechnic University, Hung Hom, Kowloon, Hong Kong, China

**Keywords:** Settlement prediction, Tunneling, Artificial intelligence-based analysis, Dataset

## Abstract

The dataset presented in this article pertains to records of shield tunneling-induced ground settlements in Guangzhou Metro Line No. 9. Field monitoring results obtained from both the two tunnel lines are put on display. In total, 17 principal variables affecting ground settlements are tabulated, which can be divided into two categories: geological condition parameters and shield operation parameters. Shield operation parameters are specifically provided in time series. Another value of the dataset is the consideration of karst encountered in the shield tunnel area including the karst cave height, the distance between karst cave and tunnel invert, and the karst cave treatment scheme. The dataset can be used to enrich the database of settlement caused by shield tunneling as well as to train artificial intelligence-based ground settlement prediction models. The dataset presented herein were used for the article titled “Evolutionary hybrid neural network approach to predict shield tunneling-induced ground settlements” (Zhang et al., 2020).

## Specifications Table

**Table:** 

Subject	Civil Engineering
Specific subject area	Geotechnical Engineering and Engineering Geology
Type of data	Table
How data were acquired	Geological investigation, on-site settlement monitoring, automatic transmission of shield machine operation data
Data format	Raw and analyzed
Parameters for data collection	Data monitoring and collection were required during the entire tunnel excavation.
Description of data collection	Geological investigation determines the geological conditions along the tunnel lines. Ground surface settlement markers were installed with approximately 5 m intervals along the tunnel alignment. The ground settlements in the study site were recorded every day except for inevitable circumstances. Shield machine operation data were recorded in real-time, and the average values of the operation parameters of each ring were taken.
Data source location	Maanshan Station-Liantang Station, Metro Line No. 9, Guangzhou, China.
Data accessibility	The relevant raw data can be found in the supplement excel file (DatasetForTraining.xlsx & MonitoringResults.xlsx).
Related research article	This article is submitted as companion paper of: Zhang K., Lyu, H. M., Shen, S. L., Zhou, A. N., Yin Z. Y. Evolutionary hybrid neural network approach to predict shield tunneling-induced ground settlements., Tunneling and Underground Space Technology.

## Value of the Data

• The dataset contains major factors that influence the ground settlement in shield tunneling.
• Those who focus on shield tunneling-induced ground settlements, especially considering the karst geological influence, may benefit from this dataset. Because this dataset includes the treatment of karst for ground settlement prediction.
• The dataset is useful for establishing an artificial intelligence-based model for predicting tunneling-induced ground settlements. This dataset can be conveniently reused by researchers. In particular, the dataset can be directly applied to train a single-target regression predictive model. Moreover, if applicable, the dataset can also form part of a larger engineering dataset, thereby being able to provide more information not only for training predictive models but also for understanding the mechanism of ground settlements induced by tunneling.

## 1 Data Description

The dataset in this article (see the supplement excel files) was collected in Maanshan Station-Liantang Station, Metro Line No. 9, Guangzhou, China [1]. The ground settlement filed data were divided into a training set (Table 1 in the DatasetForTraining.xlsx file) and a testing set (Table 2). The rows correspond to the 328 different monitoring markers along the tunnel centerlines where the data were collected. Besides, 17 variables describing the geological conditions and shield operation parameters that may affect ground settlement were tabulated in different columns. Moreover, the file titled “MonitoringResults.xlsx” records the tunnel excavation process and the detailed variation of shield operation parameters.

**Table 1:** Testing set for shield tunneling-induced ground settlement prediction.

*T* (KN)	CT (MN*m)	*v* (mm/min)	GP (kPa)	GV (m^3^)	FP (kPa)	TD	TV	GL (m)	BCT (m)	SCT (m)	RCT (m)	SIT (m)	RIT (m)	KH (m)	KD (m)	KTS	Settlement (mm)
9857	1.8	30	240	6.5	226.67	–0.44	0.07	3.38	3.50	4.07	0	3.15	26.15	0.00	100.00	0.5	–12.44
9798	1.3	44	190	6.5	226.67	–0.64	0.06	3.23	2.50	4.94	0	11.63	17.74	1.69	16.76	0	–5.68
8970	1	36	190	6.5	233.33	–0.61	0.06	3.19	2.75	4.69	0	12.81	16.55	0.00	100.00	0.5	–3.82
8992	1.3	43	210	6.5	133.33	0.02	0.06	3.61	1.73	5.61	0	4.16	25.13	0.00	100.00	0.5	-7.36
8751	1.2	38	230	7	136.67	0.03	0.01	3.25	1.39	5.94	0	1.90	27.37	0.00	100.00	0.5	–4.03
10200	2.5	28	400	6	206.67	-0.35	–0.05	3.40	2.60	4.46	0	19.64	9.49	0.00	100.00	0.5	–57.40
9700	2.3	28	400	6	173.33	-0.31	–0.02	4.25	4.23	2.77	0	6.33	22.76	0.00	100.00	0.5	–33.90
10258	2	25	300	6.5	163.33	-0.23	0.01	3.95	3.26	3.99	0	5.34	23.73	0.00	100.00	0.5	–43.30
9700	2.1	38	400	6	153.33	-0.21	0.01	3.20	2.39	5.43	0	4.17	24.86	0.00	100.00	0.5	–53.82
7300	1.6	42	400	6	123.33	0.11	–0.03	3.73	2.93	3.82	0	8.07	20.89	3.00	10.00	1	–32.20
9988	1.4	35	250	6.5	166.67	0.09	0.04	3.48	1.56	5.82	0	1.60	27.25	16.33	5.26	1	–16.30
9300	1.5	20	400	6	130.00	0.02	–0.04	3.14	3.50	3.91	0	8.98	19.80	6.20	9.28	1	–17.70
7900	1.7	42	400	6	156.67	0.28	0.02	3.35	2.94	4.30	0	4.10	24.55	1.00	7.68	0	–31.93
9999	1	35	250	6.5	143.33	0.50	0.02	3.65	2.49	4.84	0	4.59	24.02	0.00	100.00	0.5	–4.50
8700	1.5	30	400	6	143.33	0.60	0.01	3.55	1.83	5.55	0	1.95	26.59	1.30	2.45	1	–8.30
9200	1.7	40	400	6	143.33	0.64	–0.01	3.26	2.09	5.64	0	7.28	21.02	0.00	100.00	0.5	–6.26
7890	0.9	40	250	6	130.00	0.38	0.01	4.20	1.98	5.77	0	2.07	25.98	0.00	100.00	0.5	–1.93
8200	0.68	40	300	6	183.33	0.44	–0.18	3.42	2.62	4.44	0	1.84	26.05	0.00	100.00	0.5	–2.10
8780	1.4	50	250	6.5	153.33	0.54	0.02	4.23	3.10	4.92	0	8.60	19.07	1.50	9.86	1	–59.32
8980	1.4	43	250	6.5	140.00	0.54	0.03	4.21	3.47	4.52	0	11.02	16.62	0.50	10.00	1	–64.21
8540	1.4	41	250	6.5	150.00	0.52	0.03	4.35	4.20	3.82	0	17.58	10.03	0.00	100.00	0.5	–55.80
8250	1.7	72	400	7	146.67	0.50	–0.06	4.51	2.65	5.51	0	8.60	18.95	0.00	100.00	0.5	–39.74
8230	1.7	72	400	7	150.00	0.50	–0.06	3.19	2.34	5.91	0	7.02	20.48	0.00	100.00	0.5	–34.04
7900	1.5	69	400	6	150.00	0.52	–0.07	3.92	2.79	5.25	0	2.66	24.81	0.20	14.00	0	–41.02
7600	1.7	52	400	6	153.33	0.59	–0.12	4.16	2.43	5.61	0	5.80	21.64	0.00	100.00	0.5	–36.87
7600	1.7	52	400	6	153.33	0.59	–0.12	3.06	1.93	6.49	0	12.22	15.18	0.00	100.00	0.5	–33.49
7669	1.5	29	200	8	133.33	0.29	0.07	2.25	2.38	5.94	0	0.00	28.09	0.00	100.00	0.5	–6.18
8126	1.4	24	200	6	123.33	0.15	0.06	2.41	1.33	6.85	0	0.00	28.29	0.00	100.00	0.5	–1.16
9586	1.7	30	210	7	196.67	0.15	0.06	2.38	1.26	6.77	0	0.00	28.39	0.00	100.00	0.5	–2.10
8832	2.7	20	200	7	166.67	0.15	0.07	2.52	1.11	6.53	0	0.00	28.66	0.00	100.00	0.5	–6.11
8765	1.7	22	210	7	153.33	0.18	0.04	2.45	1.40	6.14	0	0.00	28.76	0.70	1.55	0	–3.76
9015	3	20	250	7	140.00	0.20	0.05	2.32	1.17	6.48	0	0.00	28.81	0.00	100.00	0.5	–2.19
12137	3.4	20	200	7	146.67	0.12	0.17	2.06	1.30	6.65	0	0.00	28.95	0.00	100.00	0.5	–2.11
12160	1.8	12	210	7	156.67	-0.02	0.05	3.43	3.11	4.10	0	2.89	26.23	0.00	100.00	0.5	–1.78
9054	1	24	200	6	133.33	-0.30	0.08	3.16	2.58	4.34	0	0.04	29.24	0.00	100.00	0.5	5.77
9800	1.8	30	300	6	126.67	–0.37	0.07	3.32	1.01	5.99	0	19.84	9.53	0.00	100.00	0.5	–13.11
10000	1.9	36	210	7	123.33	–0.52	0.13	3.33	1.08	5.90	0	8.52	20.86	0.00	100.00	0.5	–27.09
9010	1.6	30	200	6	123.33	–1.04	0.09	3.28	1.31	5.70	0	5.29	23.97	0.00	100.00	0.5	–3.82
9784	1.7	36	210	6	140.00	–1.02	0.04	3.00	1.40	5.61	0	1.99	27.26	0.00	100.00	0.5	–3.41
9672	1.7	37	200	6	143.33	–0.99	–0.09	3.17	2.14	4.86	0	10.78	18.45	0.00	100.00	0.5	–6.92
9000	1.1	30	210	6	143.33	–0.99	0.02	3.79	1.90	5.07	0	16.06	13.16	0.00	100.00	0.5	–37.85
9000	1.2	35	230	7	150.00	–0.96	0.04	3.53	2.61	4.35	0	7.13	22.08	0.00	100.00	0.5	–24.10
8211	1.1	36	210	7	146.67	–0.94	–0.07	3.26	2.79	4.19	0	10.89	18.30	0.00	100.00	0.5	–19.08
9500	2	30	210	6	143.33	–1.01	0.06	3.58	2.80	4.27	0	11.19	17.95	0.00	100.00	0.5	–3.86
12300	2.5	25	260	7	136.67	–0.66	0.05	4.10	4.33	2.82	0	16.77	12.26	2.85	21.30	0	1.11
12200	2.6	25	200	6	160.00	–0.58	0.06	3.41	2.80	4.41	0	10.58	18.38	1.60	15.08	0	–14.05
10267	2.7	32	200	6	166.67	–0.33	0.08	3.23	2.43	4.85	0	3.52	25.38	0.50	7.00	1	–13.17
9023	2.7	30	200	6	133.33	–0.06	0.04	3.15	2.40	4.91	0	13.90	14.95	0.00	100.00	0.5	–26.06
9870	2.5	35	200	6	156.67	0.28	0.03	3.80	3.70	3.63	0	9.02	19.75	5.99	9.19	1	–65.00
9053	1.5	38	220	6	156.67	0.20	0.00	3.38	3.12	4.10	0	7.68	20.96	0.00	100.00	0.5	–27.17
9037	1.4	40	300	6	163.33	0.06	0.05	3.85	3.81	3.40	0	5.60	22.99	0.00	100.00	0.5	–44.58
10980	2.4	40	200	6	160.00	0.21	0.09	4.17	4.00	3.33	0	9.47	18.95	0.00	100.00	0.5	–39.52
10781	1.8	46	200	6	156.67	0.17	0.05	3.39	4.11	3.24	0	7.56	20.72	0.00	100.00	0.5	–9.60
11487	1.4	15	220	6	160.00	0.23	0.11	5.20	4.70	2.68	0	11.32	16.90	0.00	100.00	0.5	–8.94
8802	1.7	31	200	6	123.33	–0.22	0.06	3.90	3.00	4.65	0	0.00	27.98	0.00	100.00	0.5	–12.30
10723	1.7	47	200	6	106.67	–0.16	0.09	4.60	3.03	4.65	0	0.00	27.92	0.00	100.00	0.5	–8.40
11207	2	42	200	6	163.33	0.19	0.04	3.03	2.85	4.52	0	5.36	22.94	0.00	100.00	0.5	7.40
9276	1.4	31	200	6	140.00	–1.05	-0.06	3.16	1.36	5.65	0	3.69	25.57	0.00	100.00	0.5	–3.21

The geological condition variables include groundwater level (GL), thickness of backfill over tunnel crown (BCT), thickness of sand-soil over tunnel crown (SCT), thickness of weathered rock over tunnel crown (RCT), thickness of sand-soil under tunnel invert (SIT), thickness of rock under tunnel invert (RIT), height of karst cave (KH) and distance between karst cave and tunnel invert (KD). The selected operation parameters include total thrust (T) to push the TBM during the excavation of each ring, cutter head torque (CT), penetration rate (V), tail void grouting pressure (GP), grouting volume (GV), face pressure (FP), tunneling deviation (TD), tail void (TV), karst cave treatment scheme (KTS). Among all the considered variables, 16 of which can be determined directly from the recorded data except KTS. In this study, we set a dummy variable to represent KTS and stipulate that KTS =1 represents karst cave with treatment, KTS =0 represents karst cave without treatment and KTS =0.5 represents no karst caves detected. It should be pointed out that TD and TV in Table 1 and Table 2 were processed using the principal component analysis (PCA). The original monitored TD and TV data storing in Table 3 and Table 4 in the supplement DatasetForTraining.xlsx file consist of several orientational values. The use of PCA is to reduce the number of interrelated variables [2, 3].

## 2 Experimental Design, Materials and Methods

The method used to acquire the geological data provided below (Table 1, 3, 4 in the DatasetForTraining.xlsx file & Table 2) mainly consists of geological survey. Geological survey determines the ground information of the tunnel and its surroundings, including soil type and soil mechanical properties, groundwater level, karst, etc. Basically, geological survey includes the on-site investigation for the ground information, and the indoor experiment for soil mechanical properties. The shield operation data (recorded in the MonitoringResults.xlsx file) can be automatically output by the shield machine's intelligent control system in real time. When establishing a prediction model, the operation data of the shield machines will be averaged in each ring and then used as the input data of the model.

The original monitored TD and TV consist of several orientation values. The PCA algorithm [2] is used to reduce the data redundancy. The main steps for PCA include: (1) to standardize the indicator data; (2) to find the covariance matrix of the standardized dataset; (3) to compute the eigenvalues and eigenvectors of the covariance matrix; (4) to determine the principal components. **Algorithm 1** displays the calculation process of the PCA algorithm. The PCA algorithm has been implemented in the Fortran code (see the supplement Fortran code file).

**Algorithm 1:** The calculation process of the PCA algorithm [2].

**Table:** 

**Input**: dataset $\left\{x^{n},n=1,...,N\right\}$, where *N* is the number of samples, $\dim(x^{n})=D$, *D* is the number of features.
1: Standardize the indicator data
(i) Compute the mean feature vector: $A=\frac{1}{N}\sum_{n=1}^{N}x^{n}$
(ii) Decentralize the dataset: $X^{n}=x^{n}-A$
2: Find the covariance matrix ***C*** of the standardized dataset
$\;C=\frac{1}{N-1}\sum_{n=1}^{N}X^{n}{\left(X^{n}\right)}^{T}$
3: Compute the eigenvalues $\lambda_{i}$ and eigenvectors $e_{i}$of the covariance matrix
(i) The Jacobian method can be used to solve the characteristic equation: $Ce_{i}=\lambda_{i}e_{i}$, where *i* = 1,…,D
4: Determine the principal components
(i) Determine the number of principal components *K, K* < *D*
(ii) Select the eigenvectors corresponding to the largest *K* eigenvalues to form matrix ***P***
(iii) Compute the principal component matrix: $Z^{n}={\left(P^{j}\right)}^{T}X^{n}$, where *j* = 1,…,*K*
**Output**: low dimensional dataset $\left\{Z^{n},n=1,...,N\right\}$with $\dim(Z^{n})=K$.

## Declaration of Competing Interest

The authors declare that they have no known competing financial interests or personal relationships that could have appeared to influence the work reported in this paper.

## Appendix Supplementary Materials

Image, application 1
